# Signs and symptoms of disordered eating in pregnancy: a Delphi consensus study

**DOI:** 10.1186/s12884-018-1849-3

**Published:** 2018-06-26

**Authors:** Amy Jean Bannatyne, Roger Hughes, Peta Stapleton, Bruce Watt, Kristen MacKenzie-Shalders

**Affiliations:** 1 School of Psychology, Bond University, 14 University Drive, Robina, QLD 4229 Australia; 2 Faculty of Health Sciences and Medicine, Bond University, 14 University Drive, Robina, QLD 4229 Australia; 3 School of Medicine, University of Tasmania, 17 Liverpool Street, Hobart, TAS 7001 Australia

**Keywords:** Disordered eating, Eating disorders, Pregnancy, Antenatal, Definition, Distinction, Delphi

## Abstract

**Background:**

This study aimed to establish consensus on the expression and distinction of disordered eating in pregnancy to improve awareness across various health professions and inform the development of a pregnancy-specific assessment instrument.

**Methods:**

A three-round modified Delphi method was used with two independent panels. International clinicians and researchers with extensive knowledge on and/or clinical experience with eating disorders formed the first panel and were recruited using structured selection criteria. Women who identified with a lived experience of disordered eating in pregnancy formed the second panel and were recruited via expressions of interest from study advertising on pregnancy forums and social media platforms. A systematic search of academic and grey literature produced 200 sources which were used to pre-populate the Round I questionnaire. Additional items were included in Round II based on panel feedback in Round I. Consensus was defined as 75% agreement on an item.

**Results:**

Of the 102 items presented to the 26 professional panel members and 15 consumer panel members, 75 reached consensus across both panels. Both panels clearly identified signs and symptoms of disordered eating in pregnancy and endorsed a number of clinical features practitioners should consider when delineating disordered eating symptomatically from normative pregnancy experiences.

**Conclusion:**

A list of signs and symptoms in consensus was identified. The areas of collective agreement may be used to guide clinicians in clinical practice, aid the development of psychometric tools to detect/assess pregnancy-specific disordered eating, in addition to serving as starting point for the development of a core outcome set to measure disordered eating in pregnancy.

## Background

Disordered eating has typically been defined as a range of unhealthy eating behaviours and cognitions that negatively impact an individual’s emotional, social, and physical wellbeing [1, 2]. The distinction between disordered eating and a threshold eating disorder (ED) is often the degree of severity and frequency of symptomatology, with disordered eating occurring at a lesser frequency and/or lower level of severity [2]. Much work has been done to understand the symptomatology of disordered eating in a non-pregnant context; however, the presentation and manifestation of disordered eating in pregnancy is less clear. The focus of this Delphi study was to improve clarity around the signs and symptoms of disordered eating in pregnancy, and how these can be differentiated from normative pregnancy-related changes. Such findings may assist in improving the identification of disordered eating in pregnancy.

Disordered eating in pregnancy has been linked to numerous negative consequences, such as miscarriage, prematurity, low birth weight, increased need for caesarean section, and other obstetric and postpartum difficulties [3, 4]. Adjusting to the morphological, endocrinological, and psychological changes in pregnancy, combined with the age-related vulnerability of developing disordered eating during a woman’s prime childbearing years [5–8], places pregnancy as a period of increased risk for the onset, resurgence, or exacerbation of disordered eating symptomatology, even for women with no history of such symptoms [9–19].

Over the past two decades, studies have estimated the prevalence of disordered eating in pregnancy is between 0.6 and 27.8% [12, 17, 20–23]. It is plausible, however, that existing rates under- or over- estimate the prevalence of such symptoms due to the clinical overlap between symptoms disordered eating and the experience of pregnancy, and the absence of pregnancy-specific disordered eating psychometric instruments [12]. In addition to representing a persistent pattern of disturbance, disordered eating can also represent changes in eating and exercise patterns due to developmental stages (e.g., pregnancy, early childhood, and advancing age), other mental health conditions (e.g., major depressive disorder), or certain life events (e.g., moving away from home, relationship breakdown). In these circumstances, the changes in an individual’s eating and/or exercise patterns are typically transient and/or not accompanied by significant psychological or physical distress [2].

In relation to pregnancy, most women report disturbances in normal eating patterns [18], usually in the form of food cravings, increases or decreases in appetite, changes to dietary preferences, inconsistent eating patterns, food aversions, and nausea and vomiting [24, 25]. Despite these behaviours being normal within the context of pregnancy due to hormonal fluctuations, changes in sensory perception, and maternal and/or fetal nutritional needs [26], many of these pregnancy-appropriate changes overlap with, and could possibly mask, disordered eating symptomatology [12]. For example, ‘eating for two’ could be confused with binge eating, persistent pregnancy sickness could be explained by purging, and changes in dietary preferences and/or reduced appetite could be equated to dietary restriction. A further barrier for identification of disordered eating in pregnancy is introduced when volitional stigma is considered, with research suggesting women experiencing disordered eating in pregnancy are reluctant to disclose their symptoms due to fear of stigma [27–30]. Frontline antenatal practitioners (e.g., midwives/nurses, obstetricians, and general practitioners [GPs]), in addition to other allied health professionals in contact with women during pregnancy (e.g., psychologists, dietitians) may therefore struggle to identify disordered eating in pregnant women, particularly when symptoms fluctuate between alleviation and exacerbation depending on the course and stage of pregnancy [31]. In many instances, clinicians also lack the required training for such identification [7].

The aim of the present Delphi study was to obtain subject matter expert consensus on the expression and distinction of disordered eating in pregnancy to improve awareness and understanding of such symptoms across various health professions (e.g., obstetrics, midwifery/nursing, general practice, psychology, dietetics, exercise physiology, and physiotherapy) and at a community level, in addition to informing the development a pregnancy-specific assessment instrument that may assist in facilitating early identification.

## Methods

**Fig. 1:**
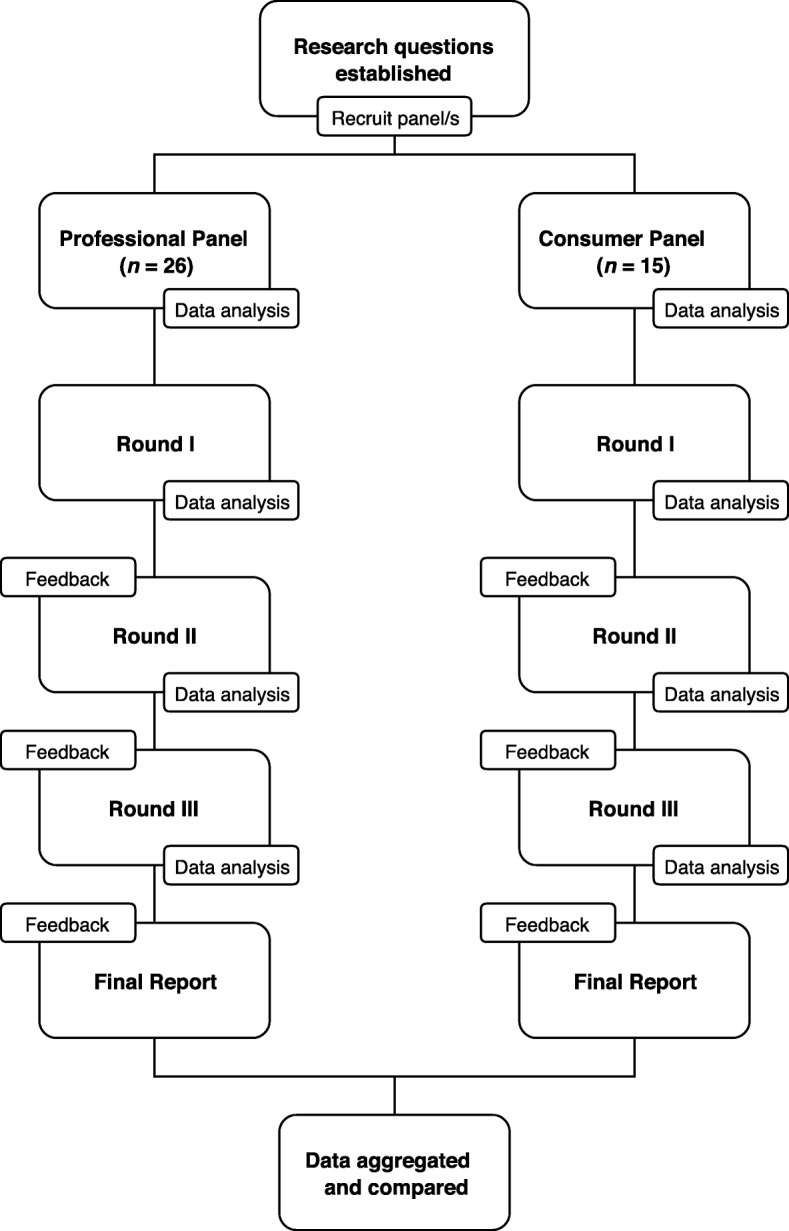
Overview of the Delphi process in the current study

The present study used a modified Delphi method [32–34]; a formal methodology used in a range of fields and settings to facilitate consensus discussions among a group of experts when accepted knowledge about a topic/issue/definition is absent or limited [35]. In a broad sense, the Delphi method involves several iterative questionnaires (rounds) to canvass and organise the opinions of an anonymous group of individual experts (panellists). The panel moderator provides structured feedback in between each round to elicit ongoing reflection, usually summaries of the quantitative results and qualitative themes from the previous rounds. This multi-stage procedure continues until a certain level of consensus is reached [33] or, in more recent years, a ‘stop’ criterion is met [36]. The process used is shown in Fig. 1.

### Participants (Panellists)

Two independent Delphi panels were recruited to ensure diverse opinions could be generated and all perspectives considered. International clinicians and researchers with expertise in the field of disordered eating, particularly in relation to pregnancy and/or women’s health (i.e., professionals) formed one panel. The other panel consisted of women who identified with a lived experience of disordered eating in pregnancy (i.e., consumers). Panel recruitment and data collection was approved by the Bond University Human Research Ethics Committee (#15278) in Australia.

Professionals in the current study met one of the following criteria: (a) established interest and expertise in the treatment of disordered eating, preferably within the context of the perinatal period, and/or women’s health; (b) distinguished contribution to the field of EDs as evidenced by (i) Fellowship status by the Academy for Eating Disorders (AED), (ii) Associate Professor or Professor in the field of EDs and/or women’s health, (iii) more than 10 years experience working in the field of EDs and/or women’s health, or (iv) publication of peer-reviewed journal article(s) and/or book(s) focused on EDs/disordered eating and/or women’s health in the perinatal period. Researchers were identified through authorship of relevant articles during a systematic review of literature, and clinicians were identified via online searches, membership of special interest groups, and professional network suggestions. AED Fellows with relevant clinical or research interests, as listed on the AED website, were also contacted. Potential professional panel members were invited to participate in the study via an email that outlined the rationale and purpose of the study, how the results would be used, and the procedure of a Delphi study. It was also noted the study would be carried out in English. Of the 80 emails sent, there was a 44% response rate, which is similar to other published Delphi studies on the topic of EDs [37–39].

Unlike recruitment for the professional panel, it was not possible to employ purposive invitation-based sampling for the consumer panel due to ethical reasons. As such, expression of interest recruitment was utilised, similar to other Delphi studies [40]. This was achieved by posting advertisements on online pregnancy and parenting forums (e.g., BubHub, Raising Children Network, Essential Baby, and Huggies), in addition to targeted advertising on social media platforms (e.g., Facebook, Twitter). Women who identified with an experience of disordered eating in pregnancy, and were interested in participating in the study, were asked to contact the primary researcher and briefly detail their experience. This primarily occurred via email. As one of the main aims of the Delphi process was to clarify the symptomatology of disordered eating in pregnancy, the inclusion criteria for the consumer panel were broad. During the pre-screening process, if a woman described eating-, body image-, or exercise-related behaviours, attitudes, or thoughts that were distressing or caused functional impairment during pregnancy, an invitation to participate was offered. Women who disclosed a medical condition that may have produced such symptoms (e.g., hyperemesis gravidarum) were not invited to participate. Women were invited to participate regardless of symptoms being active or inactive at the time of recruitment. Of the 22 consumers who were invited to participate, there was an 86.4% participation rate.

### Procedure

Data were collected across three questionnaire rounds between March and November 2016 using a secure, online survey platform (Qualtrics). Professional and consumer panellists were given four to 5 weeks to complete each questionnaire round, with reminder emails sent twice during each questionnaire completion period. In a systematic review of 100 Delphi studies, Diamond et al. [41] revealed the median threshold for determining consensus was 75% (range: 50 to 97%). As such, consensus in the current study was defined as at least 75% agreement (i.e., ratings of *important* and *very important*, or *agree* and *strongly agree*) on an individual item. All items were rated at least twice (i.e., in Rounds I and II) prior to the decision to include (≥ 75% agreement), re-rate in Round III (50–74% agreement) or remove (< 50% agreement). Items suggested at the end of Round I were automatically rated in Rounds II and III to obtain two rounds of data. Items were evaluated independently in each panel, and then compared at the end of the study.

#### Round I

Consistent with a modified Delphi method, a comprehensive literature search of both academic and grey literature was conducted between October and December 2015 to inform the content of the initial questionnaire. Key search terms were used to locate relevant websites, journal articles, reports, clinical guidelines, books (including diagnostic criteria), booklets, and training manuals. Consistent with Bond et al. [42], the grey literature search was conducted using Google Australia, Google UK, Google USA, and Google Books, while the academic literature search was performed using PubMed and PsycINFO databases. The key search terms used were: (eating disorders OR disordered eating) in pregnancy; (manage* OR support* OR treat*) (disordered eating OR eating disorders) in pregnancy; (defining OR symptoms of) disordered eating in pregnancy; (screening OR assessment OR identification) of (disordered eating OR eating disorders) in (pregnancy OR antenatal OR perinatal OR maternity care).

The first 50 items in each search were retrieved and reviewed for relevance, after duplicate sources were removed [42–44]. To minimise the influence of searching algorithms on Google, as recommended by Bond et al. [42], several steps were undertaken: (a) the history in Google’s search settings was routinely cleared to minimise the influence of previous searches, (b) care was taken to ensure the primary researcher was not logged into any Google-related accounts (e.g., Gmail) that may utilise demographic details to target searches or information; (c) location features that may bias information presented were disabled and the ‘any country’ function on Google’s searches was de-selected to ensure only local pages in each search region were shown. Sources were included if they were in English, related to EDs/disordered eating specifically in the context of pregnancy, and addressed the key areas under consideration. Pertinent information from each source was categorised thematically according to the areas of investigation in a spreadsheet by the primary researcher. When a search hit generated a website landing page with multiple hyperlinks, all links were reviewed. Overall, 200 sources were used to develop the Round I questionnaire (see Table 1).

**Table 1:** Summary of Sources that Contributed to the Development of the Round I Questionnaire

Source type	Number included	Example/s
Websites (general educational materials, pamphlets, news articles, forums)	72	https://www.thewomens.org.au/health-information/pregnancy-and-birth/mental-health-pregnancy/eating-disorders-in-after-pregnancy/http://www.cci.health.wa.gov.au/docs/ACF383.pdf
Empirical journal articles	84	Easter et al. (2013) [12], Tierney et al. (2013) [59]
Clinical guidelines or reports	18	National Eating Disorders Collaboration (2015) [16]
Conference proceedings	3	Burton (2014) [60]
Theses	6	Tremblay (2015) [61]
Books	17	American Psychiatric Association (2013) [1] Franko (2006) [62]

The primary researcher met with each member of the research team on several occasions to finalise the Round I questionnaire, which resulted in three main sections. Each section included a brief summary of existing literature to contextualise the items that followed. The purpose of these summaries was not to prime panellists in responding, but to present a rationale for why rating of such items was necessary. Throughout the study, panellists were encouraged to draw upon their own experiences when responding to each item. In section one, panellists were asked to indicate the extent to which they agreed that an item reflected a sign or symptom of disordered eating in pregnancy on a 5-point Likert scale (1 = *strongly disagree* to 5 = *strongly agree*). A total of 61 symptoms were presented to both panels for rating in Round I. In section two, panellists were asked to indicate how important certain factors were in distinguishing disordered eating from pregnancy-appropriate symptomatology (foci items) on a 5-point Likert scale (1 = *not important* to 5 = *very important*). A total of 32 foci items were presented to both panels for rating in Round I. Assessment patterns and methods were assessed in section three; the results of this section are presented in Bannatyne et al. [45].

To allow rich data to emerge for subsequent questionnaire round development, open-ended questions were included in the Round I questionnaire to facilitate and elicit feedback and suggestions for additional items in each section. Round II and III also included open-ended text boxes; however, use of these was limited to panellists contextualising responses (if required) or providing feedback to the panel moderator if there was difficulty answering a question. Prior to administration, the final version of the Round I questionnaire was piloted on 10 colleagues unconnected to the study (5 academic researchers and 5 clinicians) to ensure adequate face and content validity.

#### Round II

Responses from the Round I questionnaire were pooled and analysed in SPSS Version 23 using measures of central tendency (mean and mode), dispersion (standard deviation), and frequency. Panel comments elicited from the open-ended text boxes were downloaded and transferred into a Word processing document and analysed using thematic analysis. Common themes were identified and grouped together, and cross-coded by two independent researchers to ensure accuracy. These comments were then translated into new quantitative items to be included in Round II, provided the ideas had not been included in the Round I questionnaire and were relevant to the scope of the project. It should be noted that although the professional and consumer panel were recruited concurrently, there was a delay in receiving the Round I responses of four consumer panel members due to technology difficulties. To prevent significant attrition from the professional panel, the decision was made to send out the Round II questionnaire for the professional panel, while waiting for the consumer responses to be returned. The outcome of this decision was that Round I item suggestions from the professional panel (8 new symptom items, 1 new foci item) could be incorporated into the Round II questionnaire of both the professional and consumer panel; however, the Round I item suggestions from the consumer panel (20 new symptom items, 1 new foci item) could only be incorporated into the Round II questionnaire of the consumer panel (i.e., the professional panel did not rate new items suggested by the consumer panel). This also meant that items ratings were evaluated independently in each panel. In other words, the two panels operated independently of each other until the end of the study when items that reached consensus in both panels were compared.

Administration of the Round II questionnaire was identical in terms of instruction and format to the Round I questionnaire; however, the Round II questionnaire included a summary of the group results from Round I at the beginning of each section. This summary included both central tendency scores for each item and a summary of qualitative feedback. Items that reached the 75% consensus agreement threshold were highlighted for panellists using bolding and asterisks.

#### Round III

A similar collation and analysis process was performed on the data from Round II. Administration of the Round III questionnaire followed the same format as the Round II questionnaire. No new symptom or foci items were introduced in Round III; however, panellists were asked to determine the broad frequency at which symptoms might be considered ‘disordered’ in pregnancy. These symptoms were framed as “a significant influence of body weight and shape on self-evaluation in the presence of any compensatory behaviour aiming to prevent/reduce pregnancy-related weight gain AND/OR the presence of binge eating episodes/behaviours that occur and are followed by feelings of guilt or shame”. Frequency response options included *once per month, once per fortnight, once per week,* and *twice per week.* Panellists were asked to select one response. The purpose of this question was to identify a broad proxy that may assist clinicians to distinguish disordered eating from normative pregnancy experiences.

## Results

### Panel demographics

#### Professional panel

A total of 32 experts were recruited, with 26 completing all three rounds (81.3% retention rate). Overall, the final sample consisted of 23 women and three men from geographically diverse areas, with an average of 19.08 years (*SD* = 11.56) respective professional experience and 14.42 years (*SD* = 10.97) specialisation in the field of EDs/disordered eating. Seven panel members also identified as AED Fellows, a status that recognises distinguished contributions in the area of EDs. See Table 2 for additional panel details.

**Table 2:** Additional demographic details for the professional panel (*N* = 26)

Demographic variable	n (%)
Residing country	
Australia	12 (46.2%)
United States	6 (23.1%)
United Kingdom	4 (15.4%)
Canada	2 (7.7%)
Sweden	2 (7.7%)
Highest level of education	
Doctorate / PhD	19 (73.1%)
Masters Degree	4 (15.4%)
Postgraduate Degree (unspecified)	2 (7.7%)
Undergraduate Degree	1 (3.8%)
Professional field	
Psychology / Psychiatry	21 (80.1%)
Dietetics	4 (15.4%)
Obstetrics	2 (7.7%)
Midwifery	1 (3.8%)
Professional activities	
Researcher also involved in clinical practice	11 (42.3%)
Clinician with no research activities	8 (30.8%)
Researcher with no current clinical practice	4 (15.4%)
Clinician with some research involvement	2 (7.7%)
Other	1 (3.8%)

#### Consumer panel

A total of 19 women were recruited, with 15 completing all three rounds (79.0% retention rate). The age of the final sample ranged from 23 to 43 years (*M* = 45.62 years, *SD* = 12.08), with the majority of Caucasian ethnicity (86.6%). Of the final sample, five women were pregnant at the time of recruitment (31.2%), one had recently given birth within the past 6 months (6.3%), one had given birth within the past year (6.3%), seven had given birth within the past 2 years (43.8%), and one had given birth within the past 3 years (6.3%). In exploring the pregnancy that disordered eating was experienced in, 10 women (66.7%) reported an experience of disordered eating in only one pregnancy, with 70% noting this was experienced in their first pregnancy (*n* = 7). Five women (33.3%) reported experiences of disordered eating in multiple pregnancies, including their first pregnancy. For most of the panel, disordered eating was experienced during a planned pregnancy (80.0%). Of the five women who were pregnant during the study, all had given birth previously and all reported experiencing disordered eating in their previous and current pregnancy.

### Section 1: Signs and symptoms of disordered eating in pregnancy

Overall, 48 of the 69 potential attributes rated across both panels reached the consensus agreement criterion, including behavioural (22 of 27), physical (3 of 14), cognitive (13 of 16), and affective (10 of 12) symptomatology. An additional 20 items were generated and rated only by the consumer panel, with 19 reaching the consensus threshold. See Table 3 for a list of all the symptom attributes. Both panels endorsed a similar number of behavioural, cognitive, and affective symptom attributes; however, the professional panel endorsed a greater number of physical symptom attributes compared to the consumer panel (10 vs 3, respectively). Cohen’s kappa (κ) was performed to determine endorsement agreement between the two panels. Results differed depending on the symptom category under consideration, with poor agreement on physical symptoms (κ = .165) but very strong agreement on behavioural symptoms (κ = .867). Overall, agreement on all symptoms was modest (κ = .467).

**Table 3:** Panel ratings for the potential symptom attributes of disordered eating in pregnancy

	Panel	Mean (*SD*)	Mode	% of panel agreement	Consensus
Behavioural symptom items					
Dietary consumption that does not support a healthy pregnancy	P	4.88 (.33)	5.00	100%	Yes
	C	4.67 (1.05)	5.00	93.3%	Yes
Dieting behaviours (e.g., calorie counting)	P	4.15 (.68)	4.00	92.3%	Yes
	C	4.13 (1.41)	5.00	80.0%	Yes
Inflexibility and rigidity with diet (i.e., strict consumption of diet foods only)	P	4.88 (.33)	5.00	100%	Yes
	C	4.07 (1.03)	4.00	86.7%	Yes
Fasting and/or skipping meals	P	4.88 (.33)	5.00	100%	Yes
	C	4.53 (1.06)	5.00	93.3%	Yes
Use of meal replacements (when not advised by a health professional)	P	4.54 (.81)	5.00	88.5%	Yes
	C	4.40 (1.40)	5.00	86.7%	Yes
Repeated weighing	P	3.85 (.78)	4.00	76.9%	Yes
	C	4.67 (1.05)	5.00	93.3%	Yes
Refusing to eat outside of one’s home	P	4.65 (.56)	5.00	96.2%	Yes
	C	4.33 (1.23)	5.00	86.7%	Yes
Eating in secret	P	4.73 (.45)	5.00	100%	Yes
	C	4.60 (1.06)	5.00	93.3%	Yes
Eating an objectively large amount of food	P	3.85 (.54)	4.00	76.9%	Yes
	C	3.93 (1.03)	4.00	80.0%	Yes
Eating for “two”	P	2.46 (.76)	2.00	7.7%	No
	C	3.33 (.72)	3.00	33.3%	No
Eating when not physically hungry	P	3.08 (.56)	3.00	19.2%	No
	C	4.13 (.52)	4.00	93.3%	Yes
Using food to cope with/soothe strong emotions, or reward oneself	P	3.92 (.63)	4.00	84.6%	Yes
	C	4.07 (1.10)	4.00	80.0%	Yes
Eating rapidly and until uncomfortably full	P	4.31 (.62)	4.00	92.3%	Yes
	C	4.13 (1.13)	5.00	80.0%	Yes
Self-induced vomiting	P	4.85 (.46)	5.00	96.2%	Yes
	C	4.53 (1.13)	5.00	86.7%	Yes
Obsessively exercising for the purpose of controlling weight and shape	P	4.15 (.54)	4.00	92.3%	Yes
	C	4.60 (1.06)	5.00	93.3%	Yes
Exercising against medical recommendations	P	4.92 (.27)	5.00	100%	Yes
	C	4.60 (1.06)	5.00	93.3%	Yes
Exercising in secret	P	4.88 (.33)	5.00	100%	Yes
	C	4.80 (.56)	5.00	93.3%	Yes
Refusing to purchase maternity clothing	P	2.96 (.82)	3.00	15.4%	No
	C	2.93 (1.22)	2.00	33.3%	No
Wearing specific clothing to conceal pregnancy	P	2.88 (.71)	3.00	15.4%	No
	C	3.67 (1.05)	4.00	68.8%	No
Misuse of gestational diabetes medication	P	4.96 (.20)	5.00	100%	Yes
	C	4.80 (.78)	5.00	93.3%	Yes
Use of laxatives or enemas to reduce gestational weight gain/induce weight loss	P	4.92 (.27)	5.00	100%	Yes
	C	4.80 (.78)	5.00	93.3%	Yes
Use of appetite suppressants or “diet pills”	P	4.88 (.43)	5.00	96.2%	Yes
	C	4.80 (.78)	5.00	93.3%	Yes
Use of natural supplements (e.g., tea detox)	P	4.81 (.49)	5.00	96.2%	Yes
	C	4.67 (.82)	5.00	93.3%	Yes
Body checking behaviours	P	4.00 (.49)	4.00	88.5%	Yes
	C	4.80 (.41)	5.00	100%	Yes
Self-harm	P	4.85 (.37)	5.00	100%	Yes
	C	4.40 (.91)	5.00	86.7%	Yes
Not consuming enough food during pregnancy to produce milk or sustain breastfeeding, resulting in weight loss and/or binge eating behaviours ^a^	P	4.87 (.34)	5.00	100%	Yes
	C	4.60 (.63)	5.00	93.3%	Yes
Spending an excessive amount of time (i.e., multiple hours per week) researching about the most effective ways to reduce pregnancy weight gain and/or ways to lose weight after birth	P	–	–	–	–
	C	4.93 (.26)	5.00	100%	Yes
Searching for or seeking information about disordered eating in pregnancy	P	–	–	–	–
	C	4.53 (.92)	5.00	86.7%	Yes
Using the pregnancy as a ‘valid’ excuse/reason to avoid feared foods and/or not violate dietary rules	P	–	–	–	–
	C	4.53 (.52)	5.00	100%	Yes
Obsessively recording anticipated and achieved weight gain and calculating calorie intake and exercise output to ensure only the absolute minimum weight gain (and feeling distressed if anything interferes with this)	P	–	–	–	–
	C	5.00 (.00)	5.00	100%	Yes
Preferring to ensure the nausea and ignore physical hunger signals due to fear of weight gain or changes to shape	P	–	–	–	–
	C	5.00 (.00)	5.00	100%	Yes
Going to bed hungry at the end of the day and thinking about food, but not allowing oneself to eat to subside this hunger	P	–	–	–	–
	C	4.93 (.26)	5.00	100%	Yes
Excessively reassuring doctors/midwives that low weight during pregnancy OR lack of weight gain is nothing to be concerned about by reporting vague eating habits (e.g., “I eat heaps”)	P	–	–	–	–
	C	4.80 (.78)	5.00	93.3%	Yes
Requesting early discharge from hospital because of the food that might be served and feeling anxious is this early discharge does not or cannot occur	P	–	–	–	–
	C	4.73 (.59)	5.00	93.3%	Yes
Frequent ‘fat talk’ (i.e., if a pregnant woman talks a lot about how ‘fat’ she looks or is)	P	–	–	–	–
	C	4.40 (.91)	5.00	86.7%	Yes
Chewing and spitting out large amounts of food, particularly forbidden foods	P	–	–	–	–
	C	4.93 (.26)	5.00	100%	Yes
Physical symptom items					
Low body weight	P	3.96 (.53)	4.00	96.2%	Yes
	C	3.80 (.56)	4.00	73.3%	No
Losing weight while pregnant	P	4.73 (.60)	5.00	92.3%	Yes
	C	3.80 (.68)	4.00	80.0%	Yes
Inadequate gestational weight gain	P	4.77 (.65)	5.00	96.2%	Yes
	C	4.46 (.64)	5.00	93.3%	Yes
Excessive gestational weight gain	P	3.88 (.65)	4.00	80.8%	Yes
	C	3.80 (.68)	4.00	66.7%	No
Rapid gestational weight gain	P	3.92 (.56)	4.00	80.8%	Yes
	C	3.60 (.83)	4.00	66.7%	No
Dizziness and/or fatigue	P	3.54 (.76)	3.00	46.2%	No
	C	2.93 (.80)	3.00	13.3%	No
Feeling nauseated most of the time	P	2.08 (.85)	2.00	7.7%	No
	C	2.67 (1.18)	3.00	20.0%	No
Severe morning sickness that does not stop after the first trimester (hyperemesis gravidarum)	P	4.31 (.84)	5.00	84.6%	Yes
	C	2.00 (1.36)	1.00	20.0%	No
Dehydration	P	4.58 (.58)	5.00	96.2%	Yes
	C	3.27 (.80)	3.00	33.3%	No
Abdominal bloating	P	3.04 (.60)	3.00	11.5%	No
	C	2.93 (.80)	3.00	13.3%	No
Gastrointestinal discomfort	P	3.00 (.63)	3.00	19.2%	No
	C	2.47 (.99)	2.00	13.3%	No
Unborn baby is small/underdeveloped for gestational age ^a^	P	3.96 (.48)	4.00	87.0%	Yes
	C	3.47 (.74)	3.00	33.3%	No
Asymmetrical or slow foetal growth ^a^	P	3.96 (.48)	4.00	87.0%	Yes
	C	3.53 (.74)	3.00	40.0%	No
The woman’s blood tests show electrolyte imbalances (e.g., low potassium) ^a^	P	4.31 (.84)	5.00	84.6%	Yes
	C	4.13 (.74)	4.00	80.0%	Yes
Cognitive symptom items					
Overvaluation of body shape and weight	P	4.42 (.50)	4.00	100%	Yes
	C	4.93 (.26)	5.00	100%	Yes
Perceptual disturbance (e.g., perceiving self to be overweight for pregnancy stage, when objectively not)	P	4.42 (.50)	4.00	100%	Yes
	C	4.87 (.35)	5.00	100%	Yes
Poor body image	P	4.12 (.52)	4.00	92.3%	Yes
	C	4.47 (.99)	5.00	80.0%	Yes
Low self-esteem	P	3.77 (.65)	4.00	73.0%	No
	C	4.20 (.56)	4.00	93.3%	Yes
Rumination about gestational weight gain	P	4.04 (.53)	4.00	88.5%	Yes
	C	4.87 (.35)	5.00	100%	Yes
Rumination about health of baby	P	3.08 (.63)	3.00	15.4%	No
	C	3.07 (.80)	3.00	20.0%	No
Fixation on post-partum weight loss	P	4.12 (.52)	4.00	92.3%	Yes
	C	4.80 (.56)	5.00	93.3%	Yes
Self critical thoughts and fear of criticism	P	3.31 (.79)	3.00	42.3%	No
	C	4.20 (.56)	4.00	93.3%	Yes
Comparing personal eating habits to others	P	3.77 (.59)	4.00	76.9%	Yes
	C	3.87 (.74)	4.00	80.0%	Yes
Need for pregnancy to be “perfect”	P	3.88 (.71)	4.00	76.9%	Yes
	C	4.20 (.78)	4.00	93.3%	Yes
Desire for baby to be “small” or “petite”	P	4.73 (.53)	5.00	96.2%	Yes
	C	4.20 (1.08)	5.00	80.0%	Yes
Suicidal thoughts/ideation	P	4.62 (.94)	5.00	88.5%	Yes
	C	4.40 (.83)	5.00	80.0%	Yes
Frequent comparison of weight and shape, with pregnant and non-pregnant women ^a^	P	4.74 (.45)	5.00	100%	Yes
	C	4.67 (.62)	5.00	93.3%	Yes
Belief that vomiting will not adversely impact the fetus/baby because “all pregnant women vomit” ^a^	P	4.78 (.52)	5.00	96.0%	Yes
	C	4.60 (.74)	5.00	86.7%	Yes
Obsessive thoughts during pregnancy that relate to food (e.g., fear of food contamination, “clean eating” to avoid pesticides) ^a^	P	4.74 (.45)	5.00	100%	Yes
	C	4.47 (.83)	5.00	93.3%	Yes
Obsessive thoughts regarding health and normality of pregnancy ^a^	P	3.96 (.64)	4.00	87.0%	Yes
	C	4.07 (.85)	4.00	80.0%	Yes
Thoughts during pregnancy about using breastfeeding as a purgatory method and/or prolonging breastfeeding for weight loss	P	–	–	–	–
	C	4.73 (.80)	5.00	93.3%	Yes
Agonising and debating the absolute necessity of every food item consumed and/or bargaining with oneself	P	–	–	–	–
	C	4.93 (.26)	5.00	100%	Yes
Urges and thoughts of wanting to vomit to relieve physical or psychological tension	P	–	–	–	–
	C	2.93 (1.22)	2.00	33.3%	No
Thoughts that one does not deserve to eat, and having to justify food consumption ‘for the baby’	P	–	–	–	–
	C	5.00 (.00)	5.00	100%	Yes
Thoughts of wanting to be ‘just bump’ (i.e., weight gain is only acceptable in ‘pregnancy-appropriate’ areas such as the stomach, but not the arms/thighs etc)	P	–	–	–	–
	C	4.33 (.82)	4.00, 5.00	93.3%	Yes
Thoughts of returning to a restrictive diet once the baby is no longer dependent on mother’s body (e.g., to grow in the womb, for breastfeeding, etc)	P	–	–	–	–
	C	4.60 (.74)	5.00	86.7%	Yes
Preoccupation with diets, weight management information, and the lack of weight gained by other pregnant individuals and/or admiration for how rapidly these individuals ‘snap back’ to their pre-pregnancy body weight and shape	P	–	–	–	–
	C	4.93 (.26)	5.00	100%	Yes
Affective symptom items					
Distress regarding changing shape + fear of fatness	P	4.27 (.45)	4.00	100%	Yes
	C	4.53 (1.06)	5.00	86.7%	Yes
Distress or guilt after eating “unhealthy” or “bad” foods	P	4.19 (.49)	4.00	96.2%	Yes
	C	4.53 (.83)	5.00	93.3%	Yes
Mood disturbance	P	3.92 (.80)	4.00	84.6%	Yes
	C	3.13 (.99)	3.00	33.3%	No
Anxiety about certain foods/food groups	P	4.08 (.56)	4.00	84.6%	Yes
	C	4.67 (.49)	5.00	100%	Yes
Feeling “out of control” of one’s body	P	4.27 (.45)	4.00	100%	Yes
	C	4.60 (.91)	5.00	86.7%	Yes
Feeling a “loss of control” over eating	P	4.77 (.59)	5.00	92.3%	Yes
	C	4.53 (1.06)	5.00	93.3%	Yes
Guilt after eating (any food)	P	4.35 (.49)	4.00	100%	Yes
	C	4.73 (.46)	5.00	100%	Yes
Feelings of shame + disgust about body	P	4.92 (.27)	5.00	100%	Yes
	C	4.80 (.41)	5.00	100%	Yes
Sensitivity to comments regarding weight, shape, or appearance	P	4.04 (.60)	4.00	92.3%	Yes
	C	4.20 (.94)	5.00	80.0%	Yes
Emotional detachment from pregnancy	P	4.46 (.86)	5.00	84.6%	Yes
	C	4.27 (.82)	4.00	80.0%	Yes
Social isolation	P	4.31 (.97)	5.00	84.6%	Yes
	C	4.47 (.74)	5.00	86.7%	Yes
Interpersonal mistrust	P	3.73 (.72)	4.00	76.9%	Yes
	C	3.73 (.88)	4.00	73.3%	No
Feeling relieved or thankful for pregnancy serving as a valid explanation to avoid certain foods or eating very little	P	–	–	–	–
	C	4.87 (.35)	5.00	100%	Yes
Distress in relation to increased appetite during pregnancy	P	–	–	–	–
	C	4.87 (.35)	5.00	100%	Yes
Feeling resentful toward the baby for needing constant food and nutrients to grow in the womb, followed by significant guilt and shame for feeling resentful	P	–	–	–	–
	C	4.60 (1.12)	5.00	93.3%	Yes

*P* professional panel (*N* = 26), *C* consumer panel (*N* = 15) Items were rated on a 5-point Likert scale (1 = *strongly disagree* to 5 = *strongly agree*)

^a^additional item suggested by professional panel in Round I

### Section 2: Distinguishing disordered eating from pregnancy-appropriate symptoms

Overall, 27 of the 33 indicators rated across both panels to distinguish symptoms of disordered eating from pregnancy-appropriate symptomatology reached the consensus agreement criterion. One additional foci item was generated and rated only by the consumer panel, reaching consensus. Endorsement agreement between the panels was very strong (κ = 1.00). In general, there was agreement across both panels that practitioners could clarify the clinical overlap using a blend of clinical judgment, functional analysis, observation of informational discrepancies, assessment of impact and impairment, and consideration of patient and familial historical factors. The list of foci item ratings can be found in Table 4, while key quantitative and qualitative factors for clinicians to consider are shown in Table 5.

**Table 4:** Panel ratings for potential factors relevant in distinguishing disordered eating in pregnancy from pregnancy-appropriate symptomatology

*Distinguishing foci*	Panel	Mean (*SD*)	Mode	% of panel agreement	Consensus
Severity of behaviours	P	4.88 (.43)	5.00	96.2%	Yes
	C	4.80 (.41)	5.00	100%	Yes
Severity of cognitions	P	4.88 (.43)	5.00	96.2%	Yes
	C	5.00 (.00)	5.00	100%	Yes
Frequency of behaviours	P	4.85 (.46)	5.00	96.2%	Yes
	C	4.87 (.35)	5.00	100%	Yes
Frequency of cognitions	P	4.85 (.46)	5.00	96.2%	Yes
	C	5.00 (.00)	5.00	100%	Yes
Dietary behaviours in excess to recommended guidelines	P	4.46 (.71)	5.00	88.5%	Yes
	C	4.13 (.64)	4.00	86.7%	Yes
Dietary behaviours in deficit to recommended guidelines	P	4.73 (.60)	5.00	92.3%	Yes
	C	4.33 (.62)	4.00	93.3%	Yes
Exercise behaviours in excess to recommended guidelines	P	4.35 (.75)	5.00	84.6%	Yes
	C	4.33 (.49)	4.00	100%	Yes
Exercise behaviours in deficit to recommended guidelines	P	3.19 (.90)	3.00	34.6%	No
	C	3.33 (1.11)	3.00	40.0%	No
Appropriateness of gestational weight gain	P	3.96 (.45)	4.00	88.5%	Yes
	C	4.20 (.56)	4.00	93.3%	Yes
Health risk or distress to fetus	P	4.88 (.43)	5.00	96.2%	Yes
	C	5.00 (.00)	5.00	100%	Yes
Health risk or distress to mother	P	4.85 (.54)	5.00	92.3%	Yes
	C	5.00 (.00)	5.00	100%	Yes
Distress of (or worry by) family	P	3.92 (.48)	4.00	92.3%	Yes
	C	4.13 (.92)	4 /5.00	80.0%	Yes
History of pregnancy complications (e.g., miscarriage, premature labour)	P	3.96 (.48)	4.00	84.6%	Yes
	C	4.67 (.72)	5.00	86.7%	Yes
Level of physical impairment or impact	P	4.04 (.66)	4.00	88.5%	Yes
	C	4.93 (.26)	5.00	100%	Yes
Level of psychological impairment or impact (e.g., affective state of mother)	P	4.31 (.66)	4.00	92.3%	Yes
	C	5.00 (.00)	5.00	100%	Yes
Level of social impairment or impact	P	4.12 (.59)	4.00	88.5%	Yes
	C	4.93 (.26)	5.00	100%	Yes
Level of relational impairment or impact	P	4.04 (.59)	4.00	84.6%	Yes
	C	4.93 (.26)	5.00	100%	Yes
Degree of flexibility with dietary rules	P	4.58 (.58)	5.00	96.2%	Yes
	C	4.47 (.52)	4.00	100%	Yes
Level of insight and/or denial	P	4.81 (.49)	5.00	96.2%	Yes
	C	4.40 (.83)	5.00	93.3%	Yes
Discrepancy between self-reported functioning and medical observations	P	4.81 (.49)	5.00	96.2%	Yes
	C	5.00 (.00)	5.00	100%	Yes
Discrepancy between the woman’s report and partner/family reports	P	4.73 (.53)	5.00	96.2%	Yes
	C	4.73 (.46)	5.00	100%	Yes
Available coping strategies (e.g., emotion regulation skills)	P	4.00 (.63)	4.00	88.5%	Yes
	C	4.80 (.41)	5.00	100%	Yes
Available social support	P	4.92 (.69)	4.00	92.3%	Yes
	C	4.73 (.46)	5.00	100%	Yes
History of any psychiatric condition	P	4.08 (.69)	4.00	88.5%	Yes
	C	5.00 (.00)	5.00	100%	Yes
History of an eating disorder	P	4.85 (.46)	5.00	96.2%	Yes
	C	5.00 (.00)	5.00	100%	Yes
History of subclinical disordered eating behaviours	P	4.85 (.46)	5.00	96.2%	Yes
	C	4.93 (.26)	5.00	100%	Yes
Family history of an eating disorder	P	4.00 (.57)	4.00	92.3%	Yes
	C	4.20 (.56)	4.00	93.3%	Yes
Younger age (< 30 years)	P	2.88 (.59)	3.00	7.7%	No
	C	1.40 (1.06)	1.00	6.7%	No
Older age (> 30 years)	P	2.85 (.54)	3.00	3.8%	No
	C	1.53 (1.25)	1.00	13.3%	No
Ethnicity	P	2.73 (.67)	3.00	0.0%	No
	C	1.60 (1.12)	1.00	6.7%	No
Primigravidity (first pregnancy)	P	2.96 (.44)	3.00	7.7%	No
	C	2.20 (1.52)	1.00	20.0%	No
Multigravidity (subsequent pregnancies)	P	2.88 (.52)	3.00	3.8%	No
	C	2.13 (1.41)	1.00	20.0%	No
Ability to return to “normal eating” and regain feelings of control (w/out being restrictive) after bouts of pregnancy-related appetite changes ^a^	P	4.52 (.47)	5.00	86.9%	Yes
	C	4.73 (.53)	5.00	100%	Yes
Intent behind the behaviour (e.g., restricting one’s food intake is only problematic if the intention is to minimise weight gain or lose weight during pregnancy, as opposed to restricting due to nausea)	P	–	–	–	–
	C	4.93 (.26)	5.00	100%	Yes

*P* professional panel (*N* = 26). *C* consumer panel (*N* = 15) Items were rated on a 5-point Likert scale (1 = *not important* to 5 = *very important*)

^a^additional item suggested by professional panel in Round I

**Table 5:** Questions to consider when evaluating potential symptoms of disordered eating in pregnancy

• How often is the symptom/s occurring, and with what intensity? • What is the context and/or intent of the symptom? (e.g., *is a woman’s dietary restriction to reduce nausea or minimise gestational weight gain?)* • Does the symptom deviate from clinical recommendations during pregnancy (e.g., deficits in dietary intake, excess in exercise behaviours)? • Is the woman’s weight in a healthy range relative to pregnancy stage? Could the symptom negatively impact gestational weight gain? • Is there an actual or anticipated health risk or distress to the mother and/or unborn child? • Does a woman’s family express concern about the symptom/s? • Does the woman have a history of pregnancy complications (e.g., miscarriage, premature labour)? • Is the symptom/s causing physical, psychological, social, and/or relational impairment/difficulty for the woman? • Does the woman have insight into the presence and impact of the symptom/s? • Is the woman open to addressing the concern? • Is there a discrepancy between a woman’s self-reported functioning and the results of medical tests/observations? • Is there a discrepancy between a woman’s report of functioning and partner/family reports of functioning? • Does the woman have a history of mental health conditions, particularly eating disorders/disordered eating? • Is there a history of disordered eating in the woman’s family?	

*Note. The features in this table are reflective of the distinguishing foci that reached consensus across both panels*

**Fig. 2:**
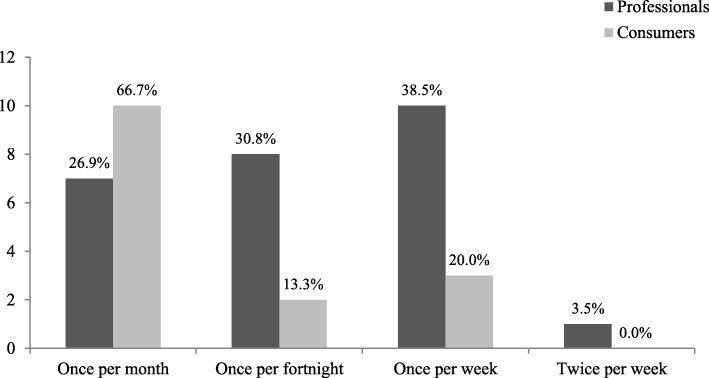
Evaluation of broad symptom frequency parameters by professionals (*n* = 26) and consumers (*n* = 15)

In terms of the broad threshold at which behaviours would be considered ‘disordered’, the most commonly endorsed response by the professional panel was weekly frequency, closely followed by fortnightly and monthly frequency. Over half the consumer panel indicated symptoms would only need to occur at least once per month to be considered problematic (see Fig. 2).

## Discussion

The present study utilised responses from professionals (clinical experts and experienced ED clinicians and researchers) and consumers (women with lived experience) to identify the signs and symptoms of disordered eating in pregnancy. Overall, the Delphi process allowed consensus to be reached between professionals and consumers on these topics.

In clarifying the manifestation of disordered eating in pregnancy, a range of behavioural, physical, cognitive, and affective signs and symptoms were identified. There was a modest level of consistency across the panels (47 symptoms meeting consensus in both panels), and generally a high level of consensus on items (31 with a consensus rate greater than 85% across both panels, 21 with a consensus rate greater than 90% across both panels). Notably, two cognitive and two affective symptoms reached 100% consensus across both panels. Cognitive symptoms were perceived to be particularly concerning by both panels given the affective distress these symptoms can produce for a woman. Such distress may have detrimental and lasting impacts on an unborn child, depending on the timing of cortisol exposure [46]. Differences in panel agreement were, however, evident for a subset of symptom attributes. In particular, the professional panel endorsed a greater number of physical symptom attributes than the consumer panel (10 vs. 3, respectively). This difference likely reflects the medical knowledge and experiences of the professional panel. As such, it may not have been appropriate to ask the consumer panel to rate such items [34].

While many of the endorsed symptoms were consistent with those likely observed in a non-pregnant context, a number of unique pregnancy-specific symptoms were endorsed across both panels including overvaluation of the offspring’s weight and shape (e.g., desire for the baby to be “small” or “petite”), rationalisation of self-induced vomiting as pregnancy-appropriate, and emotional detachment from the pregnancy. Behaviours often normalised outside of pregnancy, such as the use of natural supplements (e.g., tea detoxes) for weight loss, were also considered to be reflective of disordered eating in pregnancy and cause for concern if disclosed to clinicians practicing in this area.

Collectively, the findings suggest that practitioners working with pregnant women should be cognisant of two main factors. First, that an absence of physical or behavioural symptomatology alone does not necessarily imply a woman is unaffected by disordered eating concerns during pregnancy. Previous researchers have also suggested that while observable disordered eating behaviours often reduce during pregnancy, high levels of weight and shape concern, which cannot be easily observed and may not be disclosed freely, often persist [12, 21, 47, 48]. Second, that disordered eating in pregnancy reflects a spectrum of behaviours that do not necessarily result in physical weight or shape changes, and that particular exploration of binge eating behaviours and cognitions may be justified. Such notion supports previous work [4, 10, 11, 17]. Together these findings seem reasonable; yet, antenatal practitioners report a lack of knowledge and confidence in identifying disordered eating symptomatology [7, 29]. Furthermore, ED literature suggests that community understanding of the spectrum of disordered eating is poor, with binge eating and/or non-purgatory weight control behaviours often perceived as normative or benign [49]. To assist clinicians working in this area, the signs, symptoms, and delineating factors revealed in this study could be used as a starting point to aid identification. Results of the current study may also encourage and assist in the development of training resources to increase frontline antenatal practitioners’ (e.g., obstetricians, GPs, midwives, and nurses) and other allied health professionals’ (e.g., dietitians, psychologists, exercise physiologists, and physiotherapists) awareness, knowledge, and understanding of the expression and manifestation of disordered eating in pregnancy.

Furthermore, emphasising the finding that disordered eating is multifaceted experience is essential, not only for practitioner awareness in potential screening and detection efforts, but also when educating women who may have limited knowledge or insight in relation to disordered eating symptoms. Historically, presentations of disordered eating in pregnancy have often been labelled ‘pregorexia’ in popular media, a term describing an excessive fear of pregnancy-related weight gain and engagement in various compensatory behaviours to avoid weight or shape changes that are characteristic of a healthy pregnancy [50–52]. Given the general population is increasingly reliant on popular media sources to obtain important information regarding their health and wellbeing [53, 54], it is plausible that women experiencing symptoms inconsistent with the explanation of pregorexia may dismiss or downplay their symptoms. Health professionals interacting with pregnant women must be aware of the potential inaccuracies popular media presentations of disordered eating may result in and the need for appropriate psychoeducation to foster awareness and insight. It is also vital that popular media outlets disseminate accurate depictions of disordered eating in pregnancy to the general population to increase awareness and reduce stigma around such symptoms, which may not be visible to a woman’s social support network.

Arguably, one of the most challenging aspects of identifying disordered eating in pregnancy is distinguishing clinical features from normative pregnancy experiences [12]. While results of the current study do not entirely clarify this nuanced distinction, there was a strong level of agreement across both panels on various quantitative and qualitative factors (outlined in Table 5) that might assist practitioners evaluate concerning symptoms. Practically, information needed to assess these factors could be gathered in routine history taking, followed by more specific questioning, particularly when symptoms are explicit. When symptoms are more subtle or ambiguous, the professional panel noted implementation of clinical judgment would be required. This may include normative comparison of behaviours to clinical guidelines; evaluation of functional impairment across multiple domains; and assessment of insight/denial via observed behavioural discrepancies. In terms of the frequency at which symptoms may be considered problematic, our findings revealed the consumer panel considered symptoms of relatively low frequency (once per month) to be distressing, compared to professional panel who considered weekly frequency to be concerning. Further research is, however, required to explore/confirm this finding.

Although the current study has provided a preliminary expert-derived template for understanding and distinguishing disordered eating from pregnancy-appropriate symptomatology, there are a few limitations worth noting. First, it is acknowledged that the list of symptom attributes and delineating foci generated in the current study is not exhaustive and further discussion in this area is required. Second, as the Delphi methodology does not allow panelists to discuss topics directly with each other, it is possible that rich information often elicited from intellectual discourse with one’s peers may have been missed [33]. This could have been achieved through the implementation of a consultation meeting [55]; however, the anonymity of the panels likely prevented power-imbalances and group think that may have developed via direct contact [56, 57]. Third, although the professional panel did consist of various professions, it was difficult to recruit certain professionals, namely obstetricians, and male panellists for balanced viewpoint. There are several possible explanations for this. One likely explanation is that the schedules and unpredictable workload of obstetricians have precluded participation in a study over a six-month period; however, flexible completion options were offered to all participants. Possibly, potential panellists from the field of obstetrics may not have identified with the label ‘expert’ due to the limited knowledge of disordered eating in pregnancy. This has been revealed in previous research and may be indicative of a greater educational issue in the field [7, 29]. Future discourse in this area would benefit from a more diverse sample of professionals of both sexes who work directly with disordered eating in an antenatal setting.

Limitations of the consumer panel should also be noted. Although the value of recruiting consumers alongside professionals has been emphasised in recent literature [34], it is possible the broad criteria for selecting consumers may have affected results, particularly given a structured criteria was employed when selecting the professional panel. This may partially explain the modest agreement between the two panels for the overall questionnaire (κ = .529); however, strong agreement was demonstrated on sections that did not rely heavily on technical knowledge, potentially suggesting that some of the discrepancies between panels (e.g., physical symptoms) were more representative of knowledge, rather than attitudinal differences. If ratings for the physical symptoms were removed, there was good agreement between the panels (κ = .672). Future research may wish to develop more specific consumer recruitment criteria based on the findings of this study, while also ensuring all viewpoints are considered. The timing discrepancy in administering the Delphi questionnaire rounds between the two panels was also undesirable, as this meant new items suggested by the consumer panel at the end of Round I could not be incorporated into the Round II questionnaire for the professional panel. Furthermore, this discrepancy precluded the possibility of evaluating items across both panels during the study. As such, the only outcome was to compare the findings of the two independent panels at the end of the study. Future research may benefit from combining consumers and professionals into a single panel (provided questions are appropriate and do not rely on specialist knowledge), or at least ensure concurrent administration of both panels to facilitate feedback and item evaluation across both panels during the Delphi process.

## Conclusions

To conclude, the areas of collective agreement in this study could guide clinicians in identifying and delineating disordered eating from pregnancy-appropriate symptomatology. It is hoped that results of this study will assist the development of psychometric tools to detect/assess pregnancy-specific disordered eating, in addition to serving as starting point for the development of a core outcome set to measure disordered eating in pregnancy [58]. This could encourage a unified research approach when measuring disordered eating symptomatology in the perinatal context and present opportunities for antenatal clinicians to provide appropriate care and support when concerning symptoms are identified.

## Abbreviations

AED: Academy for eating disorders
ED: Eating disorder
GPs: General practitioners
UK: United Kingdom
USA: United States of America

## References

[1] American Psychiatric Association (2015). Diagnostic and statistical manual of mental disorders.

[2] National Eating Disorders Collaboration (2017). Disordered eating and dieting.

[3] Linna MS, Raevuori A, Haukka J, Suvisaari JM, Suokas JT, Gissler M (2014). Reproductive health outcomes in eating disorder. Int J Eat Disord..

[4] Watson HJ, Torgersen L, Zerwas S, Reichborn-Kjennerud T, Knoph C, Stoltenberg C (2014). Eating disorders, pregnancy, and the postpartum period: findings from the Norwegian mother and child cohort study (MoBa). Nor Epidemiol.

[5] Abebe DS, Lien L, von Soest T (2012). The development of bulimic symptoms from adolescence to young adulthood in females and males: a population-based longitudinal cohort study. Int J Eat Disord..

[6] Hsu LG (1989). The gender gap in eating disorders: why are the eating disorders more common among women?. Clin Psychol Rev.

[7] Leddy MA, Jones C, Morgan MA, Schulkin J (2009). Eating disorders and obstetric-gynecologic care. J Women's Health.

[8] Stice E, Marti CN, Rohde P (2013). Prevalence, incidence, impairment, and course of the proposed dsm-5 eating disorder diagnoses in an 8-year prospective community study of young women. J Abnorm Psychol.

[9] Andersen AE, Ryan GL (2009). Eating disorders in the obstetric and gynecologic patient population. Obstet Gynecol.

[10] Knoph Berg C, Torgersen L, Von Holle A, Harmer RM, Bulik CM, Reichborn-Kjennerud T (2011). Factors associated with binge eating disorder in pregnancy. Int J Eat Disord.

[11] Bulik CM, Von Holle A, Hamer R, Knoph Berg C, Torgersen L, Magnus P, Stoltenberg C, Siega-Riz A, Sullivan P, Reichborn-Kjennerud T (2007). Patterns of remission, continuation and incidence of broadly defined eating disorders during early pregnancy in the Norwegian mother and child cohort study (MoBa). Psychol Med.

[12] Easter A, Bye A, Taborelli E, Corfield F, Schmidt U, Treasure J, Micali N (2013). Recognising the symptoms: how common are eating disorders in pregnancy?. Eur Eat Disord Rev.

[13] Harris AA (2010). Prenatal advice for caring for women with eating disorders during the perinatal period. J Midwifery Womens Health.

[14] Hawkins LK, Gottlieb B (2013). Screening for eating disorders in pregnancy: how uniform screening during a high risk period could minimise under-recognition. J Women's Health.

[15] Knoph C, Von Holle A, Zerwas S, Torgersen L, Tambs K, Stoltenberg C, Bulik CM, Reichborn-Kjennerud T (2013). Course and predictors of maternal eating disorders in the postpartum period. Int J Eat Disord..

[16] National Eating Disorders Collaboration. Pregnancy and eating disorders (2015). A professional’s guide to assessment and referral.

[17] Soares RM, Nunes MA, Schmidt MA, Giacomelle A, Manzolli P, Camey S (2009). Inappropriate eating behaviours during pregnancy: prevalence and associated factors among pregnant women attending primary care in southern Brazil. Int J Eat Disord..

[18] Tiller J, Treasure J (1998). Eating disorders precipitated by pregnancy. Eur Eat Disord Rev.

[19] Ward VB (2008). Eating disorders in pregnancy. BMJ.

[20] Broussard B (2012). Psychological and behavioural traits associated with eating disorders and pregnancy: a pilot study. J Midwifery Womens Health..

[21] Micali N, Treasure J, Simonoff E (2007). Eating disorders symptoms in pregnancy: a longitudinal study of women with recent and past eating disorders and obesity. J Psychosom Res.

[22] Pettersson CB, Zandian M, Clinton D (2016). Eating disorder symptoms pre- and postpartum. Arch Womens Ment Health.

[23] Turton P, Hughes P, Bolton H, Sedgwick P (1999). Incidence and demographic correlates of eating disorder symptoms in a pregnant population. Int J Eat Disord.

[24] Dickens G, Trethowan WH (1971). Cravings and aversions during pregnancy. J Psychosom Res.

[25] Fairburn CG, Stein A, Jones R (1992). Eating habits and eating disorders during pregnancy. Psychosom Med.

[26] Orloff NC, Hormes JM (2014). Pickles and ice cream! Food cravings in pregnancy: hypotheses, preliminary evidence, and directions for future research. Front Psychol.

[27] Franko DL, Walton BE (1993). Pregnancy and eating disorders: a review and clinical implications. Int J Eat Disord..

[28] Franko DL, Spurrell EB (2000). Detection and management of eating disorders during pregnancy. Obstet Gynecol.

[29] Morgan JF (1999). Eating disorders and gynecology: knowledge and attitudes among clinicians. Acta Obstet Gynecol Scand.

[30] Newton MS, Chizawsky LL (2006). Treating vulnerable populations: the case of eating disorders during pregnancy. J Psychosom Obst Gyn.

[31] Easter A (2015). Understanding eating disorders in the antenatal and postnatal periods. Perspect.

[32] Linstone HA, Turoff M, Linstone HA, Turoff M (1975). Introduction. The Delphi method: techniques and applications.

[33] Hasson F, Keeney S, McKenna H (2000). Research guidelines for the Delphi survey technique. J Adv Nurs.

[34] Jorm AF (2015). Using the Delphi expert consensus method in mental health research. Aust N Z J Psychiatry.

[35] Sumison T (1998). The Delphi technique: an adaptive research tool. Br J Occup Ther.

[36] Holey EA, Feeley JL, Dixon J, Whittaker VJ (2007). An exploration of the use of simple statistics to measure consensus and stability in Delphi studies. BMC Med Res Methodol.

[37] Mittnacht AM, Bulik CM (2015). Best nutrition counseling practices for the treatment of anorexia nervosa: a Delphi study. Int J Eat Disord..

[38] MacFarlane L, Owens G, Del Pozo Cruz B (2016). Identifying the features of an exercise addiction: a Delphi study. J Behav Addict.

[39] Noetel M, Dawson L, Hay P, Touyz S (2017). The assessment and treatment of unhealthy exercise in adolescents with anorexia nervosa: a Delphi study to synthesize clinical knowledge. Int J Eat Disord..

[40] Ross AM, Kelly CM, Jorm AF (2014). Re-development of mental health first aid guidelines for non-suicidal self-injury: a Delphi study. BMC Psychiatry..

[41] Diamond IR, Grant RC, Feldman BM, Pencharz PB, Ling SC, Moore AM, Wales PW (2014). Defining consensus: a systematic review recommends methodologic criteria for reporting Delphi studies. J Clin Epidemiol.

[42] Bond KS, Jorm AF, Kelly CM, Kitchener BA, Morris SL, Mason RJ (2017). Considerations when providing mental health first aid to an LGBTIQ person: a Delphi study. Adv Ment Health.

[43] Kelly CM, Jorm AF, Kitchener BA, Langlands RL (2008). Development of mental health first aid guidelines for suicidal ideation and behaviour: a Delphi study. BMC Psychiatry.

[44] Langlands RL, Jorm AF, Kelly CM, Kitchener BA (2008). First aid for depression: a Delphi consensus study with consumers, carers and clinicians. J Affect Disord.

[45] Bannatyne AJ, Hughes R, Stapleton P, Watt B, MacKenzie-Shalders K. Consensus on the assessment of disordered eating in pregnancy: an international Delphi study. Arch Womens Ment Health. 2017; [Epub ahead of print] 10.1007/s00737-017-0806-x 29249043

[46] Davis EP, Sandman CA (2010). The timing of prenatal exposure to maternal cortisol and psychological stress is association with human infant cognitive development. Child Dev.

[47] Blais MA, Becker AE, Burwell RA, Flores AT, Nussbaum KM, Greenwood DN (2000). Pregnancy: outcome and impact on symptomatology in a cohort of eating-disordered women. Int J Eat Disord..

[48] Crow SJ, Agras WS, Crosby R, Halmi K, Mitchell JE (2008). Eating disorder symptoms in pregnancy: a prospective study. Int J Eat Disord..

[49] Mond JM, Hay PJ, Rodgers B, Owen C (2006). Self-recognition of disordered eating among women with bulimic-type eating disorders: a community-based study. Int J Eat Disord..

[50] Mathieu J (2009). What is pregorexia?. J Am Diet Assoc.

[51] Wallace K (2013). ‘Pregorexia’: Extreme dieting while pregnant.

[52] Hall-Flavin DK (2015). Is pregorexia for real?.

[53] Hogue MCB, Doran E, Henry DA (2012). A prompt to the web: the media and health information seeking behaviour. PLoS One.

[54] Fox S, Duggan M (2013). Health online 2013.

[55] Graefe A, Armstrong JS (2016). Comparing face-to-face meetings, nominal groups, Delphi and prediction markets on an estimation task. Int J Forecasting.

[56] Hsu CC, Sandford BA (2007). The Delphi technique: making sense of consensus. Pract Assess Res Eval.

[57] Williams M, Haverkamp BE (2010). Identifying critical competencies for psychotherapeutic practice with eating disordered clients: a Delphi study. Eat Disord.

[58] Duffy JMN, Rolph R, Gale C, Hirsch M, Khan KS, McManus RK (2017). Core outcome sets in women’s and newborn health: a systematic review. BJOG.

[59] Tierney S, McGlone C, Furber C. What can qualitative studies tell us about the experiences of women who are pregnant that have an eating disorder? Midwifery. 2013;29:542–49. 10.1016/j.midw.2012.04.013 23149238

[60] Burton T. Walking a tightrope: women's experiences of having an eating disorder while pregnant. Aust Nurs Midwifery J. 2014;21:45. 24812785

[61] Tremblay KA. Eating and psychological distress during pregnancy: The use of ecological momentary assessment (doctoral dissertation). 2015. Retrieved from ProQuest Dissertations and Theses Database (UMI No. 3732671).

[62] Franko DL. Eating disorders in pregnancy and the postpartum. In: Hendrick V, editor, Psychiatric disorders in pregnancy and the postpartum. Totowa: Humana Press; 2006. pp. 179–196.

